# Biomarker evidence of neurodegeneration in mid-life former rugby players

**DOI:** 10.1093/brain/awaf152

**Published:** 2025-07-03

**Authors:** Neil S N Graham, Karl A Zimmerman, Jessica Hain, Erin Rooney, Ying Lee, Martina Del Giovane, Thomas Parker, Mathew G Wilson, Paresh Malhotra, Michael C B David, Magdalena Kolanko, Maneesh Patel, Elena Veleva, Owen Swann, Amanda Heslegrave, Henrik Zetterberg, Daniel Friedland, Richard Sylvester, David J Sharp

**Affiliations:** UK Dementia Research Institute Centre for Care Research & Technology Centre, Imperial College London, London W12 0BZ, UK; Department of Brain Sciences, Imperial College London, London W12 0BZ, UK; Centre for Injury Studies, Imperial College London, London W12 0BZ, UK; UK Dementia Research Institute Centre for Care Research & Technology Centre, Imperial College London, London W12 0BZ, UK; Department of Brain Sciences, Imperial College London, London W12 0BZ, UK; Centre for Injury Studies, Imperial College London, London W12 0BZ, UK; UK Dementia Research Institute Centre for Care Research & Technology Centre, Imperial College London, London W12 0BZ, UK; Department of Brain Sciences, Imperial College London, London W12 0BZ, UK; UK Dementia Research Institute Centre for Care Research & Technology Centre, Imperial College London, London W12 0BZ, UK; Department of Brain Sciences, Imperial College London, London W12 0BZ, UK; Institute of Sport, Exercise and Health (ISEH), University College London, London W1T 7HA, UK; UK Dementia Research Institute Centre for Care Research & Technology Centre, Imperial College London, London W12 0BZ, UK; Department of Brain Sciences, Imperial College London, London W12 0BZ, UK; Institute of Sport, Exercise and Health (ISEH), University College London, London W1T 7HA, UK; UK Dementia Research Institute Centre for Care Research & Technology Centre, Imperial College London, London W12 0BZ, UK; Department of Brain Sciences, Imperial College London, London W12 0BZ, UK; UK Dementia Research Institute Centre for Care Research & Technology Centre, Imperial College London, London W12 0BZ, UK; Department of Brain Sciences, Imperial College London, London W12 0BZ, UK; Centre for Injury Studies, Imperial College London, London W12 0BZ, UK; Institute of Sport, Exercise and Health (ISEH), University College London, London W1T 7HA, UK; HCA Healthcare, HCA Healthcare Research Institute, London W1T 7HA, UK; UK Dementia Research Institute Centre for Care Research & Technology Centre, Imperial College London, London W12 0BZ, UK; Department of Brain Sciences, Imperial College London, London W12 0BZ, UK; UK Dementia Research Institute Centre for Care Research & Technology Centre, Imperial College London, London W12 0BZ, UK; Department of Brain Sciences, Imperial College London, London W12 0BZ, UK; UK Dementia Research Institute Centre for Care Research & Technology Centre, Imperial College London, London W12 0BZ, UK; Department of Brain Sciences, Imperial College London, London W12 0BZ, UK; Department of Imaging, Imperial College Healthcare NHS Trust, London W6 8RF, UK; UCL, UK Dementia Research Institute at University College London, London WC1N 3BG, UK; Department of Neurodegenerative Disease, UCL Institute of Neurology, Queen Square, London WC1N 3BG, UK; UCL, UK Dementia Research Institute at University College London, London WC1N 3BG, UK; Department of Neurodegenerative Disease, UCL Institute of Neurology, Queen Square, London WC1N 3BG, UK; UCL, UK Dementia Research Institute at University College London, London WC1N 3BG, UK; Department of Neurodegenerative Disease, UCL Institute of Neurology, Queen Square, London WC1N 3BG, UK; UCL, UK Dementia Research Institute at University College London, London WC1N 3BG, UK; Department of Neurodegenerative Disease, UCL Institute of Neurology, Queen Square, London WC1N 3BG, UK; Department of Psychiatry and Neurochemistry, Institute of Neuroscience and Physiology, the Sahlgrenska Academy at the University of Gothenburg, Mölndal 413 90, Sweden; Clinical Neurochemistry Laboratory, Sahlgrenska University Hospital, Mölndal 413 45, Sweden; Hong Kong Center for Neurodegenerative Diseases, Clear Water Bay, Hong Kong, China; Wisconsin Alzheimer’s Disease Research Center, University of Wisconsin School of Medicine and Public Health, University of Wisconsin-Madison, Madison, WI 53792, USA; UK Dementia Research Institute Centre for Care Research & Technology Centre, Imperial College London, London W12 0BZ, UK; Department of Brain Sciences, Imperial College London, London W12 0BZ, UK; Institute of Sport, Exercise and Health (ISEH), University College London, London W1T 7HA, UK; Acute Stroke and Brain Injury Unit, National Hospital for Neurology and Neurosurgery, Queen Square, London WC1N 3BG, UK; UK Dementia Research Institute Centre for Care Research & Technology Centre, Imperial College London, London W12 0BZ, UK; Department of Brain Sciences, Imperial College London, London W12 0BZ, UK; Centre for Injury Studies, Imperial College London, London W12 0BZ, UK

**Keywords:** TBI, concussion, head injury, CTE, dementia, sports

## Abstract

Repetitive head impacts and traumatic brain injuries in contact sports, such as rugby union, are associated with increased risk of neurodegenerative diseases such as Alzheimer's disease and chronic traumatic encephalopathy. Advances in fluid and imaging biomarkers are transforming dementia diagnosis but have not been systematically applied to individuals previously exposed to head impacts during rugby participation.

We used biomarkers, including those with sensitivity and specificity for early Alzheimer's pathology to explore neurodegenerative risk in mid-life elite retired rugby players with significant repetitive head impact exposure. Plasma neurofilament light, glial fibrillary acid protein, amyloid-β (Aβ)_42_, Aβ_40_ and phospho-tau_217_ were quantified using ultrasensitive single molecule array digital enzyme-linked immunosorbent assay (SiMoA) in former elite rugby players as well as age/sex-matched unexposed controls. 3 T MRI and neuropsychology assessments were performed, with National Institute for Neurological Disorders and Stroke criteria used to ascertain the presence of traumatic encephalopathy syndrome. Regression models were used to relate plasma/imaging biomarkers to clinical phenotype. Individual-levels analyses were performed for fluid and imaging metrics, based on control biomarker distributions. Biomarker data from two aligned un-exposed Alzheimer's cohorts were included to contextualize our findings.

Two hundred ex-rugby players (median age 44 years, 90% males) and 33 unexposed controls were assessed. Twenty-four (12%) ex-players fulfilled criteria for traumatic encephalopathy syndrome but none had dementia. Plasma phospho-tau_217_ concentrations were 17.6% higher in ex-rugby players than controls (95% confidence interval 3.7–33.3, *P* = 0.047). A total of 46 (23.1%) ex-players had elevated phospho-tau_217_ at the individual level; as did 18 (9.0%) players in relation to raised plasma neurofilament light. Ex-players’ concentrations were lower than in unexposed adults with late onset Alzheimer's disease (*n* = 69). Ex-players also showed significantly reduced volumes in the frontal/cingulate cortex on voxel-based morphometry at the group level; with reduced white matter and lower hippocampal volume associated with longer career durations within ex-players. Trauma-associated white matter changes measured with diffusion tensor imaging were uncommon in ex-players (4.6%). Traumatic encephalopathy syndrome was significantly more common in ex-players with elevated phospho-tau_217_, while those with raised plasma neurofilament light had significantly more anxiety and depressive symptoms. Frontal brain volumes correlated negatively with neurofilament light (*r* = −0.21, *P* = 0.010), and hippocampal volumes correlated negatively with phospho-tau_217_ (*r* = −0.19, *P* = 0.024).

Elite rugby participation is associated with abnormal fluid and neuroimaging neurodegeneration biomarkers in mid-life. These include elevated phospho-tau_217_, which may indicate amyloid-dependent tau pathology. The results provide support for using state-of-the-art neurodegenerative biomarkers in the evaluation of long-term effects of sports head impact exposure.

## Teaser

Graham *et al.* used biomarkers to investigate the effects of repetitive head impacts and traumatic brain injury in mid-life former elite rugby players. Compared to unexposed controls, these individuals showed elevated plasma phospho-tau_217_ levels and reduced volumes in frontal and anterior cingulate cortex.

## Introduction

The long-term consequences of repetitive head impacts (RHI) and traumatic brain injury (TBI) are difficult to assess clinically.^1^ Epidemiological data show that up to a third of individuals decline late after injury, with cognitive, psychiatric and functional problems.^2-4^ Underlying this may be progressive neurodegenerative pathologies in some cases, which experimental injury^5^ and post-mortem correlation studies suggest could be triggered by brain injury.^6^ A range of pathologies are seen after trauma, which may directly influence the outcome, or indirectly do so via a reduction in cognitive reserve, which could increase susceptibility to other neuropathologies and the effects of brain ageing.^7^ The diagnosis, prognostication and treatment of these issues could be assisted by better *in vivo* tests. In this study, we aimed to describe the *in vivo* fluid and neuroimaging correlates of previous elite rugby union participation and relate these to clinical phenotype, including cognitive testing in the mid-life Advanced Brain Health Clinic (ABHC) cohort.^8^

The epidemiological relationship between head injury and dementia is clearly established^9-11^; however, the disease processes and neuropathologies underlying this are less certain and likely to be heterogeneous. Alzheimer's disease (AD) has been reported to be present at higher rates after TBI,^12^ yet this specific relationship has only been substantiated with neuropathology confirmation in a limited number of cases^13^ and not consistently so.^14^ Notably, acute severe TBI has been associated with widespread amyloid plaque pathology,^15^ but this should be considered distinct from ‘AD pathology’ *per se* [i.e. amyloid-β (Aβ) plaque and paired helical filament tau tangle pathology]. The trauma-specific neurodegenerative disease chronic traumatic encephalopathy (CTE), characterized by distinctive perivascular sulcal hyper-phosphorylated neuronal tau tangles,^16^ is associated with RHI, as opposed to TBI, but its prevalence remains unknown as there are not yet any tests to enable diagnosis in life.

Epidemiological data show that neurodegenerative disease is associated with rugby participation in particular, with a 2.7× increased risk of death due to neurodegenerative disease (including dementia) in rugby players compared with matched community controls.^17^ It is notable that symptomatic mild TBI (mTBI, also known as concussion) is the most common injury in rugby,^18^ with around 80% of players reporting a history of at least one previous injury.^19^ The concussion rate is estimated at around 18 per 1000 hours of professional play.^20^ Repetitive head impacts, which may be asymptomatic (‘non or sub-concussive hits’), are a common feature of the game.^21,22^ Professional rugby participation has previously been linked to poorer cognitive function,^23^ mental health^24^ and sleep quality,^25^ but retired players in mid-life have not previously been assessed in detail for possible early neurodegenerative disease, which may be relevant to these issues.

Post-mortem studies demonstrate a wide range of proteinopathies after RHI/TBI, including of Aβ, TAR-DNA-binding protein 43 (TDP-43), alpha-synuclein and tau, in addition to other neuropathologies such as white matter rarefaction and vascular injury.^6,26^ One specific disease after RHI is CTE, a spatially and molecularly-distinctive tauopathy.^16,27^ While most neuropathology studies have been performed in former American footballers, where cumulative head impact burden appears the most relevant determinant of CTE pathology,^28^ evidence is accumulating to associate neurodegenerative post-traumatic proteinopathies with participation in other contact sports, such as rugby union.^29^ A recent large series shows that CTE tau burden relates to the length of rugby playing career.^29^

Biomarkers of neurodegenerative pathology can now be assessed in the blood,^30^ with technologies such as SiMoA (single molecule array digital enzyme-linked immunosorbent assay) facilitating detection of low concentrations of neurodegenerative proteins that originate in the brain. In the subacute and chronic phase after RHI/TBI, blood biomarkers of neurodegeneration may be able to identify patients with early neurodegenerative disease or on-going brain injury. Plasma concentrations of phosphorylated forms of tau (such as p-tau_181_ and p-tau_217_) have high sensitivity and specificity for AD neuropathologic change, reflecting early amyloid and tau pathology.^31,32^ P-tau_217_ appears not to be raised in non-AD tauopathies such as corticobasal degeneration or progressive supranuclear palsy.^32^ Non-specific markers of brain injury, such as neurofilament light (NFL), glial fibrillary acidic protein (GFAP, an astroglial marker) and brain-derived tau, increase early after TBI and reflect the presence of neuronal and glial injury.^33^ Acute changes in these biomarkers predict progressive post-traumatic pathology, including brain volume loss, accelerated brain ageing and late functional deterioration.^34^ Previous work assessing fluid p-tau markers late after repetitive head trauma suggests elevations in p-tau_217_ or p-tau_181_ may be present and correlated with Aβ PET positivity,^35^ while a recent small study (*n* = 26 with fluid biomarkers) suggested elevated p-tau_181_ may be detectable in ex-players during mid-life.^36^

Neuroimaging can also be used to sensitively identify the possible direct and late neurodegenerative effects of sports participation: diffusion tensor imaging MRI is highly sensitive to traumatic white matter damage,^37^ and T_1_-weighted volumetric MRI quantifies neuronal loss, the end-product of neurodegenerative pathways. In active rugby players, we have identified changes in brain structure, including evidence of axonal and vascular injury, along with abnormal trajectories of white matter volume change.^38^ A separate small study (*n* = 24 players) suggested reductions in whole brain volume and hippocampi but with no clear relationship to cognitive function.^39^

The clinical syndromes associated with different chronic post-traumatic pathologies remain poorly characterized. Research diagnostic criteria attempting to describe the clinical phenotype of CTE, termed ‘traumatic encephalopathy syndrome’ (TES), have been produced^40^ but have not been tested in a rugby setting with the presence of substantial *in vivo* phenotyping or post-mortem confirmation of diagnosis. In the context of American footballers (where the criteria were largely calibrated)^10^ and professional fighters,^41^ there does appear to be a relationship between neurodegeneration biomarkers and a TES diagnosis. More broadly, it is unclear how injury would interact with a patient’s pre-existing neurodegenerative or ageing trajectory.

Here we evaluate the effects of repetitive head impacts and TBI on neurodegenerative blood biomarkers in former elite rugby players within the mid-life ABHC cohort.^8^ We compare fluid and MRI measures of neurodegeneration and injury between ex-players and controls, and in an exploratory analysis, assess the influence of APOE genotype. We test two key measures of exposure, specifically elite career duration and self-reported concussion burden. Our hypotheses were that ex-players would have (i) raised plasma NfL, GFAP and elevated p-tau_217_ or reduced Aβ42:40 ratio; (ii) brain volume reductions and reduced white matter integrity on MRI; and (iii) that these biomarkers would relate to cumulative head impact exposure burden and correlate with poorer clinical status.

## Materials and methods

### Study design

The ABHC cohort is a prospective study of retired elite professional rugby players who have self-referred due to concerns around their brain health.^8^ The aim of this study is to investigate the consequences of elite contact sport participation on brain health outcomes. These include white matter injury as measured by diffusion MRI measures, neurodegeneration as measured by volumetric MRI and fluid biomarkers of neurodegeneration and inflammation such as NfL, tau, p-tau_217_ and GFAP. The ex-players complete a clinical interview, neuropsychology and questionnaires to provide a comprehensive assessment of brain health. Ex-players are reviewed at the ABHC clinic and the National Institute for Neurological Disorders and Stroke (NINDS) Consensus Diagnostic Criteria for TES applied to each individual with clinical diagnoses recorded.

### Study population

Two hundred retired elite rugby players (male and female) and 33 controls were enrolled into the Advanced BRAIN Health Clinic study from November 2021 until October 2023. Ex-players entered the clinic by self-referral, with no requirement that players be experiencing cognitive symptoms, and ex-players could attend for a brain health check-up if desired. Recruitment was conducted in strict compliance with UK legislation and General Data Protection Regulation. All study participants provided written informed consent in accordance with the Declaration of Helsinki. Ethical approval was provided by the Camberwell and St Giles research ethics committee (ref 7/Lo/2066). Demographic information, past medical history, family history, functional status, symptoms and concerns were collected during a semi-structured interview. Head injury history was acquired using the BRAIN-Q^42^ and the Ohio State University (OSU) TBI Identification Method (OSU TBI-ID) Interview Form.^43^ Exposure to previous head trauma was defined by the number of self-reported concussions both whilst playing and outside of playing sports. Playing history was defined based on the number of years played at the elite level [equivalent to years playing either internationally or in the Premiership or Championship (men’s) or AP15s (women’s), with these leagues representing the highest level of domestic men’s and women’s play, and second highest men’s league in England]. This was established in a semi-structured interview and using publicly available online data about player history. Elite career duration was used, i.e. excluding amateur/youth participation, because the latter has been found to be both low in magnitude and highly variable.^44^ The first 200 ex-players attending the clinic who consented were analysed in accordance with pre-defined recruitment targets.

Inclusion criteria for ex-rugby players were age 30–61 years and previous elite level participation (e.g. in England Men/Women’s teams, England 7s Men/Women’s teams, Premiership, AP15s or Championship.) There were no exclusions other than inability to complete MRI (for the imaging part of the study). Healthy volunteers exclusions were any evidence of previous head trauma/concussion history (per the BRAIN-Q^42^ or Mayo classification^45^), history of substantial exposure to repetitive head impacts per the TES research diagnostic criteria, MRI contraindications, history of early onset dementia in a first degree relative (diagnosed before age 65) or family history of genetic dementia (e.g. Huntington’s disease, to exclude any rare autosomal dominant/early onset heritable dementia). The cohort sample size was previously determined, powered on the ability to detect significant differences in diffusion tensor imaging MRI findings between groups, noting that fluid biomarker data in this specific context are novel and were not available to inform sample size when the study was established.

### Neuropsychology assessments and questionnaires

Participants were asked to complete self-report questionnaires covering a range of domains such as mood, symptoms, sleep and quality of life. Informant/caregiver questionnaires were also completed. Participants then were assessed using established paper and computerized neuropsychological tests sensitive to impairments after TBI, including performance validity tests. Full details of tests used are available in the study protocol, which were chosen so as to be sensitive to impairments in the chronic phase after RHI/TBI.^8^ We used the Wechsler Memory Scale-Fourth Edition (WMS4) auditory memory score, rather than the Repeatable Battery for the Assessment of Neuropsychological Status (RBANS) delayed recall score, as we have found this to be a more sensitive measure.

### Traumatic encephalopathy syndrome determination

A consensus process (involving neurology and neuropsychology expertise) was followed to determine whether players met the 2021 NINDS criteria for TES.^40^ Briefly, for a TES research diagnosis to be made, participants need substantial repetitive head impact exposure. This was the case for all players, as an inclusion criterion. Second, participants need either to have significant neurobehavioural dysregulation (e.g. explosiveness, short-fuse, impulsivity, outbursts; assessed in the clinical interview, and for example using the self/informant Behaviour Rating Inventory of Executive Function^46^) or significant cognitive impairment (with deficits in episodic memory and/or executive functioning, substantiated using our neuropsychology test battery, and requiring impaired performance >1.5 standard deviations (SD) below that individual’s expected norm). In addition, these features must be delayed in onset after stopping RHI exposure, represent a significant change from baseline, be progressive in their course and not be fully explained by another cause.

### Fluid biomarker assessment

Venous blood was sampled peripherally and samples spun at 2500*g* for 10 min, transferred into 1 ml aliquots and frozen at −80°C. Plasma samples were analysed for NfL, GFAP, Aβ_40_ and Aβ_42_ using a Simoa-Neurology 4-Plex E assay, and p-tau_217_ using a Simoa ALZpath assay following the manufacturer’s protocol. A four-parameter logistic curve fit data reduction method was used to generate a calibration curve. Samples were run in duplicates and internal controls were used to calculate coefficients of variation (CVs). The mean coefficient of variation across the study was 10.1% for NfL, 6.9% for p-tau_217_, 5.7% for Aβ_40_, 5.5% for Aβ_42_ and 9.5% for GFAP. The mean of both samples was used for further analyses. Any result falling below the lower limit of quantification (LLoQ) for a given biomarker was re-recorded as 50% of the LLoQ (GFAP: 11.6 pg/ml; NfL 1.6 pg/ml; Aβ_42_ 4.08 pg/ml; Aβ_40_ 1.51 pg/ml; p-tau_217_ 0.06 pg/ml). This was the case for a single Aβ_40_ result only. One rugby player had an unrecordable p-tau_217_ concentration due to a technical issue. All reported results were included, with no removal of outliers at lower or upper limits. Statistical methods (see later) were used which are robust to the presence of any outliers. Analyses were conducted at the biomarker lab at the UK Dementia Research Institute at UCL.

For individual-level analyses, fluid biomarker concentrations were binarized into non-elevated/elevated using a 97.5th centile cut-off defined within the healthy control distribution for p-tau_217_, NfL and GFAP, and reduced/non-reduced based on a <2.5th centile cut-off for Aβ42:40 ratio values, again, using the healthy control distribution (using the r function Quantile, Stats package).

### *APOE* genotyping

DNA was extracted from whole blood. *APOE* Taqman genotyping assays were run on all samples, using assays for SNPs rs7412 and rs429358. The call rates for both assays were 100%. Participants were binarized as ε4 carriers if they had any ε4 allele. As the ε2/ε4 genotype is more common than the rare ε1/ε3 genotype, samples with calls at the cycle threshold (Ct) for rs429358 and rs7412 were interpreted as genotype ε2/ε4.

### MRI

Participants were scanned with the following sequences on a Siemens Skyra 3 T scanner: T_1_-weighted MPRAGE with voxel dimensions of 1 mm^3^ [160 slices, repetition time (TR) = 2300 ms, echo time (TE) = 2.98 ms, GeneRalized Autocalibrating Partially Parallel Acquisition (GRAPPA) = 2], T_2_ susceptibility weighted imaging (voxel size 0.8 × 0.6 × 1.2 mm, 120 slices, TR = 28 ms, TE = 20 ms, GRAPPA = 2), T_2_ fluid-attenuated inversion recovery (FLAIR; isotropic 1 mm^3^ voxel size, 160 slices, TR = 5000 ms, TE = 395 ms, GRAPPA = 2), diffusion MRI in 64 directions with isotropic voxel dimension of 2 mm^3^ (b = 1000 s/mm^3^, 4 b = 0 s/mm^2^, TR = 9500 ms, TE = 103 ms). All MRI scans were reported for the presence of focal lesions, such as microhaemorrhages. Cavum septum pellucidum presence was assessed by study neurologists blinded to participant information, and graded, being deemed to be present if at grade 2 or above, per established procedures.^47^

### Neuroimaging analysis

Volumetric T_1_ images were segmented and labelled using FreeSurfer. Volumes of the left and right frontal, parietal, temporal and occipital lobes were calculated using the lobe mapping of individual Desikan-Killiany regions of interest.^48^ Left and right hippocampal volumes and total ventricle volumes were also extracted. Voxel-based morphometry analysis of volumetric data was completed using the general linear model with non-parametric permutation testing (10 000) in FSL Randomise. Images were pre-processed using SPM12 to segment T_1_ images into grey and white matter, and CSF. A study template was generated using SPM DARTEL non-linear registration before affine registration of segmented images to Montreal Neurological Institute (MNI) 152 space, with normalization of volume and smoothing using an 8 mm kernel. Summary measures of brain volume were normalized for participant head-size by dividing them into total intracranial volume (grey matter + white matter + CSF). Volumetric results reported are corrected for multiple comparisons at a threshold of *P* < 0.1, reflecting priors for volume loss. Age, gender and total intracranial volume were included as covariates.

Diffusion MRI images were processed following the standard Tract-Based Spatial Statistics (TBSS) pipeline in the FMRIB Software Library (6.0.1). This includes correction for eddy current induced distortions and diffusion tensor fitting. Tensor-based registrations were performed using DTI-TK, which involves the creation of a group template using affine and non-linear diffeomorphic registrations, followed by registration of participant diffusion imaging to the template. Images were warped to 1 mm isotropic space, and the mean fractional anisotropy (FA) map produced was thresholded at 0.2 to produce a white matter skeleton. Subject FA and mean diffusivity (MD) data were projected onto the mean FA skeleton and tract level data generated using the Johns Hopkins University white matter atlas. Images were visually inspected at the brain extraction, eddy current correction and tensor registration stage. Subjects with poor quality data or un-correctable artefacts were removed.

Voxel-wise analysis of skeletonized diffusion metrics was conducted using the general linear model with non-parametric permutation testing (10 000) in FSL Randomise. Diffusion weighted voxel-wise results reported are corrected for multiple comparisons at a threshold of *P* < 0.05 with age and gender included as a nuisance covariate. Individualized analysis (region of interest based) was performed using the healthy control distribution of FA values per tract. Individuals with FA <2.5th centile were defined as being abnormally low in that region.

### Alzheimer’s disease and healthy older adult control group

To contextualize fluid biomarker results in our ex-players, we have included data from two linked studies (Minder and PCNorAD) within our institute,^49,50^ comprising people with AD and age-matched controls. All participants had no history of major neurological or psychiatric illnesses other than AD, including TBI (see Supplementary material, ‘Methods’ section for further detail). AD and older control participants gave plasma bloods samples as part of their respective wider studies, analyses were as per the ex-players and mid-life controls. Cognition was assessed with either the Addenbrooke’s Cognitive Examination (ACE) on the day of blood testing or with a Mini-Mental State Examination (MMSE) within 6 months of blood testing. For uniformity, MMSE scores were imputed from ACE.^51^

### Statistical analyses

Statistical tests were completed in the open-source software package R (4.3.1) using the Rstudio interface (2023.09.0 + 463). Distributions of variables were visually inspected. Non-normally distributed variables (e.g. biomarker concentrations) were log-transformed prior to regression; geometric means and SD of the non-transformed data are reported. All brain volumes were adjusted for head size by dividing by total intracranial volume prior to regression analysis.

Sex differences between ex-players and controls were tested using a Chi squared test with Yates’ correction, and age differences using a *t*-test. Linear regression was used to assess for differences between ex-players and controls, accounting for covariates (age and sex, in all models), for neuropsychology performance, questionnaire data, normalized brain volumes and biomarker concentrations. Binary logistic regression (glm function, R) was used to assess the relationship between fluid biomarker concentrations and the presence of traumatic encephalopathy syndrome in ex-players. The pscl package was used to compute pseudo R^2^ values from logistic regressions.

A complete cases approach was used throughout, where any data was missing (e.g. single p-tau_217_ result unavailable). Bonferroni correction was performed for neuropsychology tests (comprising six comparisons), questionnaires (four comparisons), volumetric pre-defined regions of interest (six comparisons), diffusion regions of interest (three comparisons), fluid biomarker concentrations (four comparisons: NfL, p-tau_217_, GFAP and Aβ42:40 ratio). Threshold-free cluster enhancement (TFCE) correction was used in voxel-wise analyses. Later regressions assessing mean values from these regions were not further multiple comparisons corrected. Exploratory Spearman’s correlations between biomarkers were calculated: no multiple comparisons correction was performed. Sensitivity analyses to assess for effects of educational level on neuropsychology performance, time since finishing rugby career on biomarker concentrations, and the effects of other head injury exposures, were completed (using these additional terms as regressors in the first two regressions, and excluding those individuals with additional injuries in the latter).

## Results

### Participant characteristics

**Figure 1:**
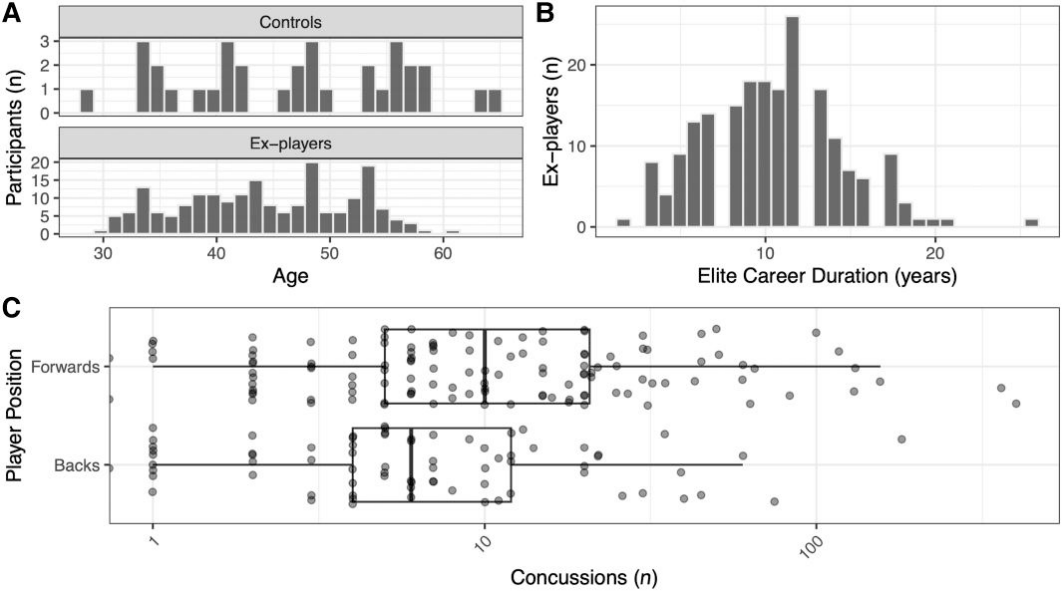
**Participant demographics, head injury history and elite career duration**. (**A**) Histogram of study participant numbers stratified by group (controls or ex-players, vertically), and by age horizontally. (**B**) Histogram showing career duration in years (elite play) within ex-players. (**C**) Box plot of concussion count (without loss of consciousness) in ex-players stratified by position.

Two hundred retired elite professional rugby players were assessed in this biomarker study, as were 33 healthy controls without repetitive head impacts nor any TBI, including mild TBI/concussion history. Demographics, trauma exposure and neuropsychology performance of the group are described in the linked publication^52^ and summarized briefly in Fig. 1 and Table 1. Ex-players had a mean age 44.3 years (SD 7.4), comprising 180 (90%) males and with a professional career duration of 10.5 years (mean, SD 4.0). The study visit was conducted an average of 13.5 years [median, interquartile range (IQR) 8.0–19] after retirement. At least one previous concussion during rugby was reported by 193 (96.5%) ex-players, with a median of seven concussions (IQR 4–20). Ex-players and controls did not differ significantly in respect of age or sex. Ex-players had higher rates of depressive and anxiety symptoms, as well as poorer scores in metacognition and behavioural regulation. Ex-players recruited into the study had no additional major neurological diagnoses.

**Table 1:** Demographics and ex-player characteristics

	Controls (*N* = 33)	Ex-players (*N* = 200)
**Demographics and exposure**		
Age at study visit, mean (SD)	46.76 (9.61)	44.34 (7.43)
Female (%)	7 (21.2)	19 (9.5)
Education in years, mean (SD)	17.03 (2.86)	15.70 (2.47)
Caucasian ethnicity, *n* (%)	32 (97.0)	190 (95.0)
*APOE* ε4 carrier, *n* (%)	11 (33.3)	60 (30.0)
*APOE* ε4 homozygote, *n* (%)	2 (6.1%)	2 (1.0%)
Player position (%)	–	–
Backs	–	74 (37.0)
Forwards	–	126 (63.0)
Elite career duration, years, mean (SD)	–	10.5 (4.0)
Retirement to assessment date, years, median [Q25,Q75]	–	13.5 [8.0, 19.0]
Concussion count, median [Q25,Q75]	–	7.0 [4.0, 20.0]^a^
Concussions with LOC, median [Q25,Q75]	–	1.0 [0.0, 3.0]
**Neuropsychology performance**		
Immediate memory (RBANS), mean (SD)	101.9 (14.1)	97.5 (14.3)
Visuospatial/constructional (RBANS), mean (SD)	115.5 (10.8)	115.4 (10.5)
Language (RBANS), mean (SD)	105.5 (10.6)	104.7 (10.9)
Attention (RBANS), mean (SD)	108.9 (13.2)	105.1 (15.4)
Processing speed (WAIS4), mean (SD)	111.7 (13.8)	105.5 (12.5)
Auditory memory (WMS4), mean (SD)	107.6 (13.3)	99.8 (14.7)
**Questionnaires**		
Depression (BDI), median [IQR]	5.50 [1.00, 9.00]	8.0 [4.0, 15.0]
Anxiety (GAD7), median [IQR]	1.00 [0.00, 4.00]	3.0 [1.0, 6.0]
Metacognition (BRIEF), median [IQR]	42.50 [39.00, 49.25]	51.0 [42.5, 61.0]
Behavioural regulation (BRIEF), median [IQR]	41.50 [38.75, 49.25]	49.0 [42.0, 58.0]
**Clinical phenotype**		
Traumatic encephalopathy syndrome, *n* (%)	–	24 (12.0)
With core cognitive impairment only, *n* (%)	–	7 (3.5)
With core neurobehavioural symptoms only, *n* (%)	–	12 (6.0)
With core cognitive and neurobehavioural problems, *n* (%)	–	5 (2.5)
TES functional dependence level		
Independent	–	19 (79.2)
Subtle/mild functional limitation	–	5 (20.1)
Mild, moderate or severe dementia	–	0 (0.0)
CTE provisional level of certainty		
Suggestive, *n*	–	21
Possible, *n*	–	3
Probable, *n*	–	0

BDI = Beck Depression Inventory; BRIEF = Behavior Rating Inventory of Executive Function; CTE = chronic traumatic encephalopathy; GAD-7 = General Anxiety Disorder-7; LOC = loss of consciousness; IQR = interquartile range; RBANS = Repeatable Battery for the Assessment of Neuropsychological Status; SD = standard deviation; TES = traumatic encephalopathy syndrome; WAIS4 = Wechsler Adult Intelligence Scale-Fourth Edition; WMS4 = Wechsler Memory Scale-Fourth Edition

^a^ *n* = 198.

In relation to non-rugby exposures, 18 (9.0%) of ex-players reported an additional exposure to RHI, which was typically martial arts (eight cases), limited-contact sport (e.g. soccer) in five cases, contact/collision sport (American football) in one case and motorsport in two cases. Four of these individuals reported a third exposure, comprising military service (two cases) and martial arts (two cases). The typical duration of participation for an additional exposure was 6.4 years (mean, SD 4.6).

Details of the Alzheimer’s disease/older controls biomarker comparison group are shown in Supplementary Table 1. Briefly, participants in the Minder/PCNorAD cohort with AD (*n* = 69) were aged an average of 75.5 years (SD 7.5), were 52% female and had a mean MMSE score of 20.8 (SD 5.3).

### Blood biomarker changes: elevated p-tau_217_ in ex-players

**Figure 2:**
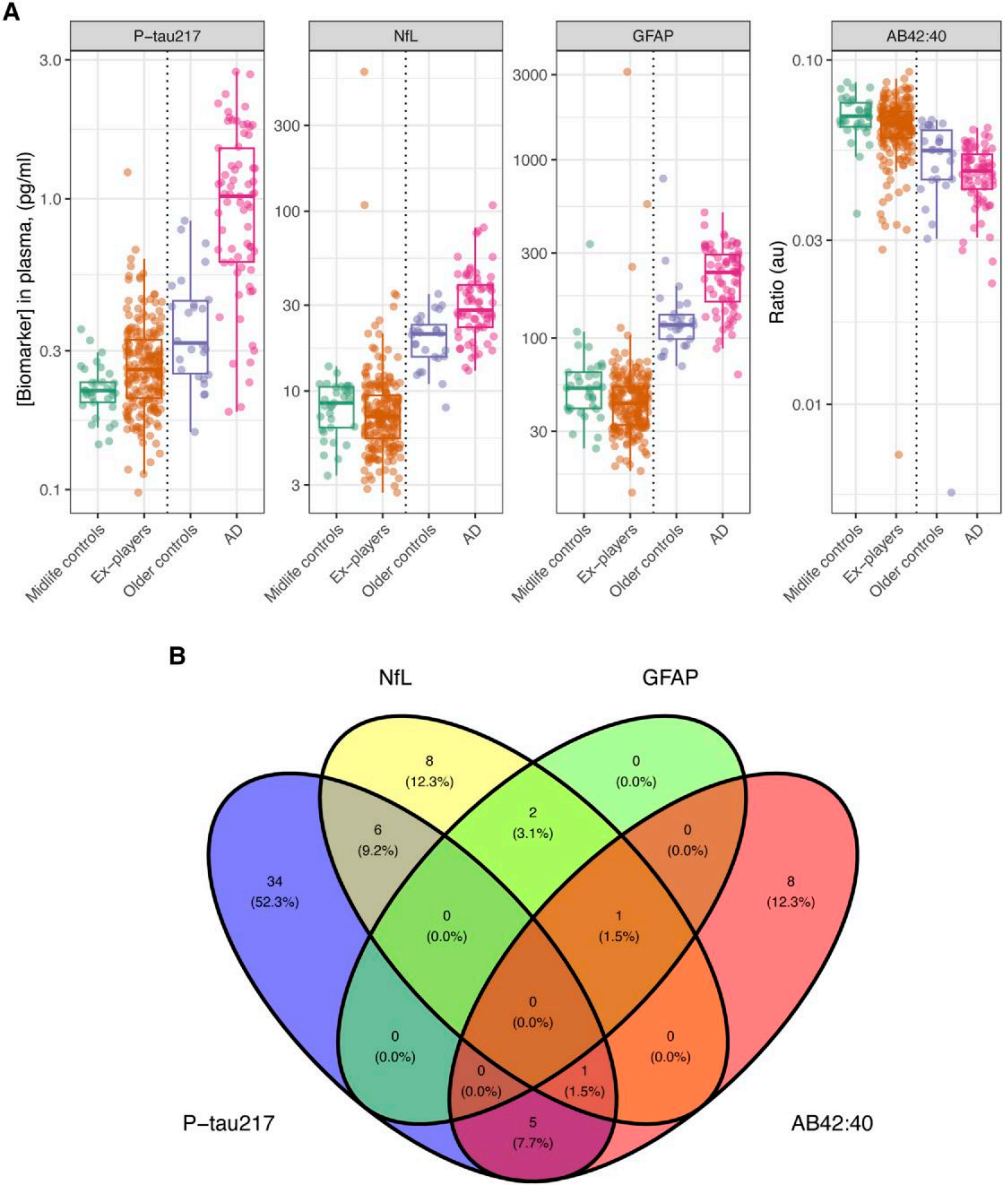
**Plasma biomarkers of neurodegeneration**. (**A**) Group level comparisons between unexposed controls, ex-players, healthy unexposed older adults, and unexposed people with late onset Alzheimer’s disease (AD). Dotted line separating ex-players/controls and AD/older controls indicates that analyses were run on the same platform with the same assay, but using different kits, hence direct comparisons and statistical tests are precluded, and groups are shown side-by-side for indicative purposes. Box plots show median and interquartile ranges with individual data points plotted. On regression, a significant group difference was present for plasma p-tau_217_ but no other biomarkers. (**B**) Venn diagram within the ex-players group only, showing individual-level abnormalities in fluid biomarkers (defined by values beyond the healthy control group 97.5th centile. Percentages shown are of all those ex-players with any biomarker abnormality (*n* = 65). Aβ42:40 ratio = amyloid-beta 42:40 ratio; GFAP = glial fibrillary acidic protein; NfL = neurofilament light; p-tau217 = phosphorylated tau 217.

Concentrations of plasma p-tau_217_ were 17.6% higher in ex-rugby players than controls [95% confidence interval (CI) 3.7–33.3, *P_corr_* = 0.047), while no significant group differences were present in plasma Aβ42:40 ratio, GFAP or NfL concentrations (Fig. 2A and Table 2). Concentrations in people with AD and older controls from the MINDER/PCNoRAD studies are described in Supplementary Table 2 (median biomarker values were numerically higher than ex-players and controls in the case of NfL, p-tau217 and GFAP, and lower in the case of the Aβ 42:40 ratio). As we expected significant variability in blood biomarker levels across ex-players, we also investigated how many individuals showed high plasma concentrations relative to the control distribution. A cut-off at the 95th percentile was calculated for elevated p-tau_217_ (0.33 pg/ml), NfL (12.8 pg/ml), GFAP (154.2 pg/ml) and for reduced Aβ42:40 ratio (0.05). At the individual level, plasma p-tau_217_ was elevated in 46 (23.1%) ex-players, defined as concentrations exceeding the 97.5th centile of the healthy control distribution. Using a similar approach, 18 (9.0%) had raised NfL and 3 (1.5%) had elevated GFAP, while the Aβ42:40 ratio was reduced in 15 (7.5%) former players. Figure 2B provides a description of the extent of overlap of these changes: of the 46 ex-players with elevated p-tau_217_, seven had elevated NfL and six had a reduced Aβ42:40 ratio, but none had elevated GFAP.

**Table 2:** Plasma biomarker concentrations in healthy controls and ex-players

	Controls	Ex-players	Group difference
**NfL, *n***	33	200	–
Concentration, pg/ml, g.mean (g.sd)	7.9 (1.4)	7.5 (1.8)	B = 1.01 (0.82–1.23), *P* = 0.949, *P_corr_* > 0.05
Elevated levels, *n* (%)	1 (3.0)	18 (9.0)	
**GFAP, *n***	33	200	–
Concentration, pg/ml, g.mean (g.sd)	53.8 (1.6)	44 (1.7)	B = 0.85 (0.71–1.02), *P* = 0.081, *P_corr_* > 0.05
Elevated levels, *n* (%)	1 (3.0)	3 (1.5)	
**P-tau_217_, *n***	33	199	–
Concentration, pg/ml, g.mean (g.sd)	0.2 (1.2)	0.3 (1.4)	**B** = **1.18 (1.04–1.33), *P*** = **0.012, *P_corr_*** = **0.047**
Elevated levels, *n* (%)	1 (3.0)	46 (23.1)	
**Aβ42:40, *n***	33	200	–
Ratio, au, g.mean (g.sd)	0.1 (1.2)	0.1 (1.3)	B = 0.91 (0.84–1), *P* = 0.04, *P_corr_* > 0.05
Reduced levels, *n* (%)	1 (3.0)	15 (7.5)	

Significant difference in corrected *P-*value for regression shown in bold. Aβ42:40 = amyloid beta 42:40 ratio; GFAP = glial fibrillary acidic protein; NfL = neurofilament light; *P_corr_* = Bonferroni-corrected *P*-value; p-tau217 = phosphorylated tau 217; sd = standard deviation.

Ex-players with elevated NfL had higher odds of greater depressive [odds ratio (OR) = 4.04, 95%CI 1.75–9.49, *P* = 0.006] and anxiety [OR = 5.36, 95%CI 2.24–13.01, *P* < 0.001] symptoms, but levels did not relate to other neuropsychology measures (Supplementary Table 3). There were no other significant correlations between blood biomarker and clinical measures. In addition, there were no significant relationships between years of elite play (i.e. career length) and concentrations of any fluid biomarker, nor was there a significant difference in biomarker concentrations related to concussion load. Furthermore, there was also no significant relationship between years of elite play and plasma biomarker concentration in a sensitivity analysis where the time between retirement and study visit (i.e. a putative ‘washout period’) was also included in the above model (Supplementary Table 4). Separately, a sensitivity analysis excluding 18 ex-players with a second RHI exposure type (see earlier) continued to show the above significant relationship between exposure (i.e. rugby versus unexposed) and plasma p-tau_217_ concentrations (B_exp_ = 1.18, 95%CI 1.04–1.34, *P* = 0.011)

### Elevated p-tau_217_ is associated with traumatic encephalopathy syndrome

We applied research criteria for TES (see the linked study in Parker *et al*.^52^). Twenty-four (12%) ex-players had a diagnosis of TES according to these criteria. Five (20.1% of the TES group) had subtle/mild limitation of function, and the remaining 19 (79.2%) were independent. No ex-rugby players had dementia. A breakdown of the TES diagnoses by core symptoms is shown in Table 1 (as are provisional levels of certainty for CTE pathology).

Within the ex-players group, elevated p-tau_217_ was associated with increased odds of TES (2.8 times more likely, 95%CI 1.1–7.1, R^2^ 0.032, *P* = 0.027) compared with players who had non-raised p-tau_217_.

In ex-players, elevated p-tau_217_ was not associated with any neuropsychological abnormalities or higher scores on symptom questionnaires. There were no significant neuropsychological correlates of reduced Aβ42:40 ratios or raised GFAP, nor did these biomarker concentrations relate to TES. A sensitivity analysis including years of education in the neuropsychology models did not influence the findings (Supplementary Table 5). Biomarker profiles in individuals with and without TES are shown in Supplementary Fig. 1.

### *APOE* ε4 and plasma p-tau_217_

We conducted an exploratory analysis of the effect of ε4 carrier status on p-tau_217_ concentration. Thirteen controls (39.4%) and 63 ex-players (31.5%) were carriers of the *APOE* ε4 allele, including two homozygous controls (6.1%) and two homozygous ex-players (1.0%). There were no significant differences in the proportion of carriers between the groups. Within the ex-players, *APOE* ε4 carriers had non-significantly higher plasma ptau_217_ (*P* = 0.065) (Supplementary Fig. 2 and Supplementary Table 6). Across the whole population (players and healthy controls), there was a non-significant trend towards higher p-tau_217_ concentrations (B = 1.10, 95%CI 1.00–1.21; *P* = 0.053) in ε4 carriers. There was no interaction between study group (i.e. ex-player or control) and carrier status in relation to p-tau concentration (*P* = 0.686). This was also the case in a sensitivity analysis conducted across the other biomarkers, where *APOE* ε4 carrier status did not significantly predict plasma concentrations, neither was there an interaction with rugby exposure, and nor did the inclusion of these terms in the model change the significance of the relationship of exposure to biomarker concentration.

### Structural brain abnormalities seen in ex-players

**Figure 3:**
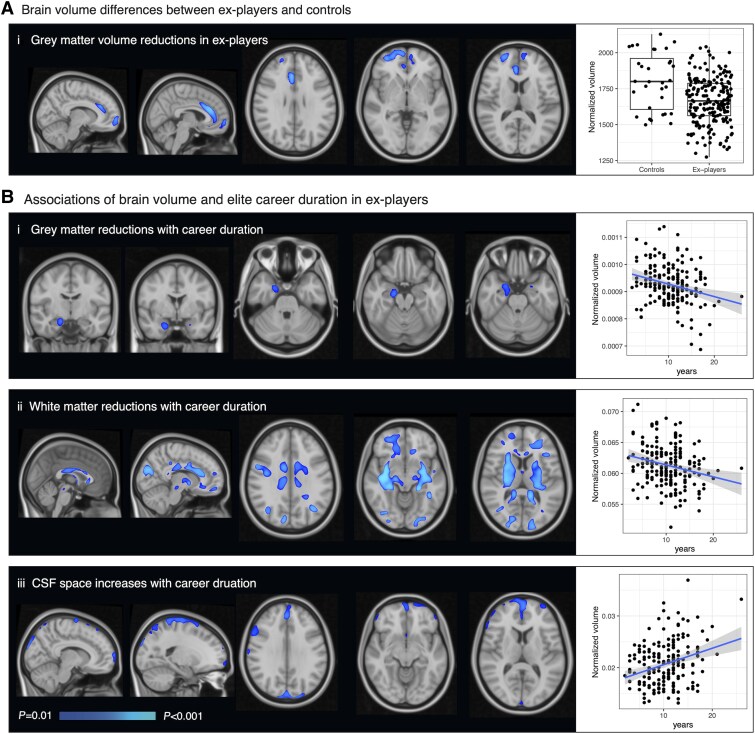
**Brain volume associations of former elite rugby participation.** [**A**(**i**)] *Left*: Voxel-based morphometry group comparison of grey matter brain volumes between ex-rugby players and healthy volunteers. TFCE-corrected significance map shown (one-tailed *t*-test; higher significance indicated by light blue colour). *Right*: Box plot showing medians and interquartile ranges of normalized grey matter volume, with individual data points plotted. [**B**(**i**)] *Left*: Significance map of regression relating career duration and lower grey matter volume within ex-rugby players. *Right*: Scatter plot showing years of play against normalized volume within this region at the individual level; linear regression line is shown in blue with 95% confidence intervals in grey. (**ii**) Significance map of regression relating career duration and lower white matter volume within ex-rugby players. *Right*: Scatter plot demonstrating the effect. (**iii**) Significance map of regression relating career duration and higher CSF volume within ex-rugby players. *Right*: Scatter plot demonstrating the effect. TFCE = threshold-free cluster enhancement.

We next investigated brain volumes using voxel-based morphometry (Fig. 3). Ex-players had significantly smaller brain volumes in the anterior cingulate cortex and superior frontal grey matter than controls (Fig. 3A). In addition, increasing career duration was associated with significant reductions of right hippocampal volume as well as widespread white matter volume reductions and CSF expansion (Fig. 3B). Years of play and concussion load in ex-players were not associated with regional volume differences (Supplementary Table 7). Within the rugby group, the volume of the anterior cingulate/frontal grey matter region identified on group voxel-based morphometry analysis was significantly reduced in individuals with TES classification (B = −108.7, 95%CI −207.3 to −10, *P* = 0.030), but other volumetric changes did not relate to this classification.

**Figure 4:**
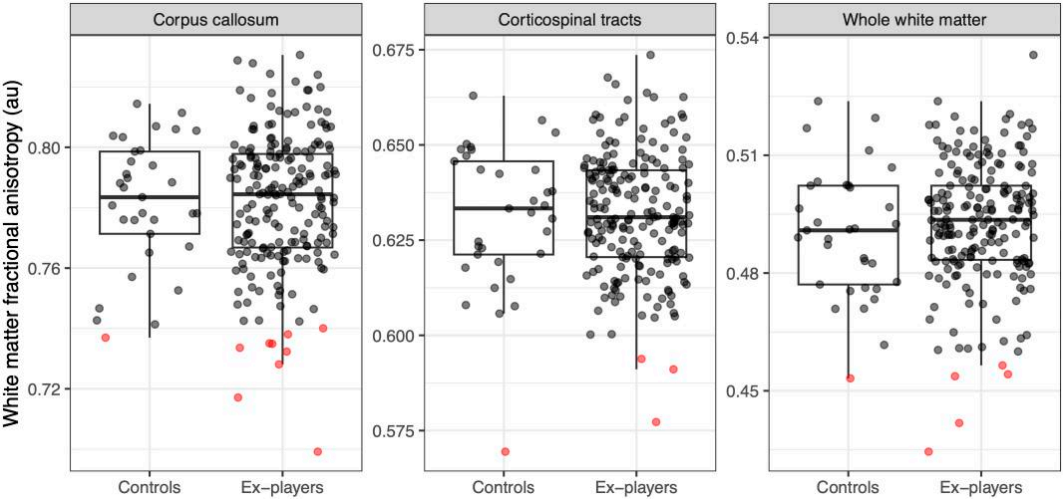
**Diffusion tensor imaging white matter microstructure in ex-players**. Fractional anisotropy values in three regions of interest in ex-rugby players and unexposed healthy volunteers, within corpus callosum (*left*), corticospinal tracts (*middle*) and whole white matter skeleton (*right*). Box plots show median and interquartile ranges with individual data points plotted. Points in red represent data below the 2.5th centile of the healthy control distribution.

We also assessed white matter tract structure using diffusion tensor imaging. Here we found no significant group differences in FA in the whole white matter or regions of interest tested (corpus callosum or corticospinal tracts) or in voxel-wise analyses (Fig. 4 and Table 3). We have previously reported abnormal FA at the individual level in around 25% of active elite rugby players.^38^ Here, we found that callosal abnormalities were present in nine (4.6%) ex-players and one (3.0%) healthy volunteer, whole white matter skeleton abnormalities in five (2.5%) ex-players versus one (3.0%) healthy volunteer and corticospinal tract abnormalities in one (3.0%) ex-player and one healthy volunteer (3.0%). There were no significant differences in the proportions of abnormal white matter tracts in ex-players and controls. FA measures did not relate to career duration or concussion load, nor was there a relationship of TES status with FA values (Supplementary Table 8).

**Table 3:** Neuroimaging characteristics

	Controls	Ex-players	Group difference
**MRI performed, *n* (%)**	33 (100)	199 (99.5)	*–*
**Cavum septum pellucidum, *n* (%)**	4 (12.1)	48 (24.0)	*P* = 0.196
**Microhaemorrhages, *n* (%)**	0 (0.0)	6 (3.0)	*P* = 0.585
**Brain volume**			
Volumetric data, *n* (%)	33 (100)	199 (99.5)	–
Frontal lobe volume, mean (SD)	0.11 (0.01)	0.11 (0.01)	B = −2029.1 (−5882.6 to 1824.3), *P* = 0.3, *P_corr_* = 1
Temporal lobe volume, mean (SD)	0.07 (0.01)	0.07 (0.00)	B = 251.7 (−2381.3 to 2884.7), *P* = 0.85, *P_corr_* = 1
Hippocampal volume, mean (SD)	0.01 (0.00)	0.01 (0.00)	B = −25.4 (−288.9 to 238.2), *P* = 0.85, *P_corr_* = 1
Parietal lobes volume, mean (SD)	0.07 (0.01)	0.07 (0.01)	B = −2210.3 (−5376.9 to 956.4), *P* = 0.17, *P_corr_* = 1
Occipital lobes volume, mean (SD)	0.03 (0.00)	0.03 (0.00)	B = −1217.8 (−3126.2 to 690.6), *P* = 0.21, *P_corr_* = 1
Ventricles volume, mean (SD)	0.01 (0.01)	0.01 (0.00)	B = −1254.7 (−4281.9 to 1772.6), *P* = 0.41, *P_corr_* = 1
**White matter microstructure**			
Usable DTI data, *n* (%)	33 (100.0)	197 (98.5)	–
Corticospinal tract FA, mean (SD)	0.63 (0.02)	0.63 (0.02)	B = −0.002 (−0.01 to 0.007), *P* = 0.699, *P_corr_* = 1
Abnormal, *n* (%)	1 (3.0)	3 (1.5)	–
Corpus callosum FA, mean (SD)	0.78 (0.02)	0.78 (0.02)	B = −0.001 (−0.007 to 0.005), *P* = 0.695, *P_corr_* = 1
Abnormal, *n* (%)	1 (3.0)	9 (4.6)	–
Whole white matter FA, mean (SD)	0.49 (0.02)	0.49 (0.02)	B = 0 (−0.006 to 0.006), *P* = 0.948, *P_corr_* = 1
Abnormal, *n* (%)	1 (3.0)	5 (2.5)	–

Diffusion normal/abnormal classification is defined by values <2.5th centile of healthy control distribution. DTI = diffusion tensor imaging; FA = fractional anisotropy; SD = standard deviation.

### NfL and p-tau_217_ correlate negatively with brain parenchymal volume in ex-players

We next investigated whether blood biomarker levels related to normalized brain volume (within frontal, parietal, hippocampal, occipital, temporal or ventricular regions) or FA change (in callosum, corticospinal tracts or whole white matter). In the group of ex-players, negative correlations were present between: plasma NfL and frontal (*r* = −0.21, *P_corr_* = 0.010) and parietal (*r* = −0.18, *P_corr_* = 0.044) volumes, p-tau_217_ and hippocampal volume (*r* = −0.19, *P_corr_* = 0.024) and GFAP with parietal volume (*r* = −0.19, *P_corr_* = 0.035). There was no significant correlation of plasma biomarkers and any diffusion tensor imaging FA metric in ex-players (Supplementary Table 9). Cavum septum pellucidum was the most frequently present abnormality on MRI, seen in 48 (24.0%) ex-players and 4 (12.1%) controls. There were no significant differences in blood biomarker concentrations in those with and without cavum septum pellucidum (Supplementary Table 10). No contusions were identified on neuroradiology review of the MRIs, although traumatic microhaemorrhages were present in six (3.0%) rugby players and no controls (Table 3).

## Discussion

The ABHC cohort of former elite rugby players in mid-life showed a number of abnormalities in biomarkers of neurodegeneration, despite no player being diagnosed with dementia. Ex-players showed significantly raised levels of plasma amyloid/tau marker p-tau_217_. Individuals with high p-tau_217_ relative to unexposed controls (23.1% of ex-players) had more chance of fulfilling criteria for TES. A significant proportion of ex-players (approaching 10%) also had elevated plasma NfL at the individual level; and group-level brain volume reductions in frontal and anterior cingulate cortex were observed in ex-players. Greater years of elite participation was associated with more hippocampal atrophy on voxel-based morphometry. The results provide support for the use of state-of-the-art neurodegenerative biomarkers in the evaluation of long-term effects of sports TBI exposure.

The observation of elevated p-tau_217_ may indicate early neurodegenerative pathology within a proportion of our ex-player cohort, as this marker of tau metabolism is raised in the earliest stages of AD. Increased p-tau_217_ is thought to reflect alteration in tau metabolism at the location of amyloid plaques and has been shown to increase prior to the development of tau PET abnormalities.^53^ Transient elevation of p-tau_217_ is seen after acute TBI,^54^ but the short half-life of changes seen after acute injury and the lack of ongoing exposure in our cohort make a direct effect of TBI unlikely. However, concentrations of p-tau_217_ in ex-players were numerically lower than in a group of older adults with late-onset AD. However, further research is needed to confirm the pathological significance of elevated p-tau_217_ in the context of mid-life sportsmen and establish robust cut points for the interpretation of plasma p-tau_217_ in this context, which may differ from those for amyloid PET positivity.

Depressive and anxiety symptoms were common in ex-players and were more prevalent in individuals with abnormally elevated NfL. While affective symptoms can be unrelated to brain diseases, they are frequently present in the early stages of neurodegenerative diseases such as Alzheimer’s and also form part of the criteria used to identify TES. NfL provides a measure of axonal pathology and neurodegeneration across a range of neurological conditions.^55^ A small number of studies in adults with major depressive disorder but without head injury history/cognitive problems have shown group-level elevations in NfL, although the mechanistic relationship between elevated NfL and neuropsychiatric symptoms is uncertain.^56-58^ Hence, it may be that increased levels of NfL indicate the presence of brain pathology that leads to neuropsychiatric problems after repetitive sports RHI/TBI. However, the magnitude of elevation is likely to be clinically important: plasma NfL can assist in differentiating fronto-temporal lobar dementia (where it is very significantly raised) from primary psychiatric disorders (PPDs),^59^ in spite of the mild elevations seen in some PPDs versus healthy controls.^60^

One important question is whether our results inform the diagnosis of CTE or other neuropathologies (including AD). Post-mortem series demonstrate that co-pathologies, including AD neuropathology, are present in individuals who have CTE pathology, and that these co-pathologies explain a substantial proportion of the clinical deficits in these individuals,^26^ while noting that the subjects in these case series are typically older than our cohort. Hence, elevated p-tau_217_ may reflect the development of preclinical AD pathology, rather than specific changes of CTE. In addition, the relationship between p-tau abnormalities and CTE pathology is uncertain. It has been reported (again, in an older study population) that individuals with CTE in the absence of AD pathology have low plasma concentrations of p-tau_217_ and p-tau_181_.^35^ However, other data suggest that p-tau abnormalities can be associated with CTE pathology in the absence of frank AD pathology. For example, post-mortem CSF in people with autopsy-confirmed with CTE showed elevated p-tau concentrations in individuals with no AD pathology compared to AD controls.^61^ Furthermore, quantification of p-tau levels within frontal tissue in patients with CTE, AD, CTE with AD and controls showed a significant decrease in p-tau_181_ in CTE versus controls, increased p-tau_231_ and p-tau_396_, as well as increased p-tau_202_, which related to the years of RHI exposure. In this analysis, all p-tau species were elevated in AD but p-tau_396_ most significantly.^62^ Our finding of hippocampal volume reductions relating to years of play has spatial similarity to previous (non-rugby) research in people with RHI exposure, where temporal atrophy was found in comparison to healthy controls.^63^

Ex-rugby players also had reduced frontal and anterior cingulate volume reductions relative to healthy controls on MRI. Our finding of frontal atrophy is consistent with limited *in vivo* assessments of patients who were later diagnosed with CTE at post-mortem.^64^ Furthermore, we found a voxel-wise association between duration of sporting career and reduced hippocampal volume, widespread white matter atrophy and CSF space expansion. These are regions which have previously been reported to have lower volumes cross-sectionally in former fighters with TES,^41^ with recent longitudinal imaging suggesting progressive accelerated hippocampal atrophy/ventricular enlargement in this context.^65^

There are several important limitations to the work. Concussion exposure was self-reported and may be subject to recall bias, although we have taken a standard approach to the retrospective ascertainment of this. It is important to note that the ex-player and control population comes from the community, with self-referral being the route for ex-players into the clinic/study, on the basis of cognitive concerns, which are relatively widespread given the publicity about the potential brain health consequences of contact sport participation. This may affect the representativeness of our participants and has implications for the study’s generalizability into other settings, such as individuals referred into memory or cognitive neurology outpatient clinics, where one might expect neuropathologies to be enriched relative to this sample. Although well powered for many analyses, we are likely to be underpowered for some analyses. In particular, the genetic analysis of *APOE* status should be viewed as exploratory and would be underpowered to detect genetic influences on clinical phenotype. Hence, the relationship between blood biomarkers and *APOE* status will require replication in larger cohorts. The control group had no history of playing professional or high-level impact sports and had no history of concussions and <5 years of RHI exposure. We cannot exclude the possibility that limited, brief exposures of controls (e.g. infrequent previous amateur participation in contact sport) may have reduced our sensitivity to the effect of RHI in ex-players. The sample size of the control group was relatively modest (*n* = 33), potentially limiting our sensitivity to detect those associates of elite rugby exposure with very small effect sizes, although this would not affect analyses within the ex-player group.

Our mid-life cohort is relatively young from the perspective of neurodegenerative risk. We diagnosed no cases of dementia, which will reflect the influence of the participants’ young age on dementia risk. It is possible that some players have an increased dementia risk that may become apparent in later decades of life. Hence, planned long-term follow-up of the cohort will be key to establishing the clinical implications of deranged fluid and imaging biomarkers at this time point, such as rates of progression (or not) to dementia. Likewise, correlation with post-mortem findings in any decedents will help to clarify the pathological basis of biomarker change, which may be distinct from typical cases of late onset AD. We were unable to formally compare fluid biomarker concentrations in this study between ex-players and the AD group, as these were undertaken using the same assay but with different kits. Future work extending and harmonizing such analyses would enable direct statistical comparisons. A further potential limitation in relation to imaging is that cavum septum pellucidum information was stratified only by grade rather than length, or other features, which could also be explored in future work. This work assessed predominantly male, Caucasian ex-players, which may limit the study’s generalizability, and where it will be particularly important to replicate the findings. Extending the study in future by including new biomarkers, such as brain-derived tau (BD-tau)^66^ and microtubule-binding region tau (MTBR-tau_243_),^67^ may better clarify the presence of neuronal/glial tau aggregates specifically, contextualizing our findings.

In conclusion, in the ABHC cohort of elite ex-rugby players, we identified significant brain volume loss on MRI and derangements in plasma neurodegeneration biomarkers, particularly the AD marker p-tau_217_. This could indicate early underlying amyloid pathology and neurodegeneration in a subset of individuals, perhaps related to amyloid-dependent neurofibrillary tau. A small number of individuals had evidence of isolated elevated plasma NfL, replicating findings in another study assessing non-rugby RHI exposures (where NfL was significantly raised in amyloid negative RHI participants),^63^ perhaps reflecting non-Alzheimer’s pathology such as CTE or TDP-43. Further work should investigate ex-players with more established diagnostics such as amyloid PET. In this mid-life cohort, there was relatively limited mapping between the clinical phenotype and biomarker profiles. TES was slightly more common in those with raised p-tau_217_, and affective symptoms were more frequently present in people with high NfL. Longitudinal follow-up of the cohort is planned to help ascertain what these biomarker changes indicate in this specific context.

## Supplementary Material

awaf152_Supplementary_Data

## Data Availability

The data that support the findings of this study are available on reasonable request from the corresponding author.
