# Supplementary material for: Biomarker evidence of neurodegeneration in mid-life former rugby players
Source: Brain. 2025 Jul 3;148(8):2684–97. doi: 10.1093/brain/awaf152 (PMC12316018; doi:10.1093/brain/awaf152)
Supplement: awaf152_Supplementary_Data [file awaf152_supplementary_data.pdf]

SUPPLEMENTARY MATEIRALS

Biomarker evidence of neurodegeneration in midlife former rugby players

Supplementary Methods

Alzheimer's disease and healthy older adult control group

A total of 69 participants with Alzheimer's disease (AD) were recruited as part of two studies: (i)'Minder', an ongoing longitudinal community-based cohort study of people with dementia run by the Care, Research and Technology Centre of the UK Dementia Research Institute[49]; and (ii) 'Physiological Correlates of Noradrenergic Add-on Therapy' (PCNorAD),[50] an experimental medicine add-on study investigating noradrenergic dysfunction in patients with AD who have taken part in the 'NorAD' clinical trial. Participants recruited from 'NorAD' underwent the procedures for this study at least one month after completing the clinical trial, except one who entered 'NorAD' after this study. 26 age-matched healthy controls were also recruited as part of 'PCNorAD'.

AD participants all had a pre-existing clinical diagnosis of AD but were also discussed within a multi-disciplinary team meeting, comprised of neurologists, psychiatrists and neuroradiologists, as part of this study. Diagnosis was based upon clinical history and investigations as well as neuroimaging and cognitive assessments conducted for research. HC were eligible for inclusion if >60 years old free from symptoms of cognitive impairment.

The Minder study was approved by the Health Research Authority's London-Surrey Borders Research Ethics Committee (19/LO/0102). The PCNorAD study was approved by the Health Research Authority's London-Central Research Ethics Committee (18/LO/0249). All participants with capacity to consent provided written informed consent for participation and for their data to be included in publications. Those without capacity were enrolled on recommendation of an assigned consultee.

Supplementary Figures

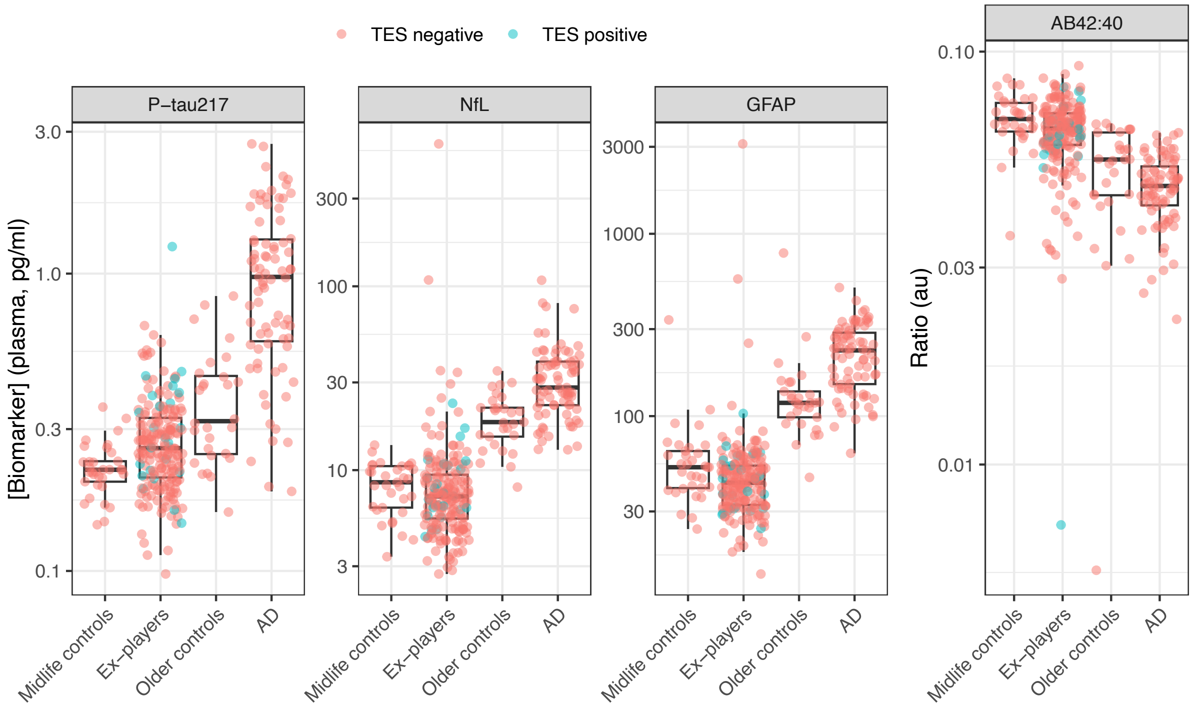

Supplementary Figure 1. Fluid biomarker levels stratified by the presence of traumatic encephalopathy syndrome *Boxplots show plasma biomarker concentrations, with median and interquartile ranges indicated, and individual data points plotted. Blue datapoints indicate TES +ve, and orange TES -ve individuals.*

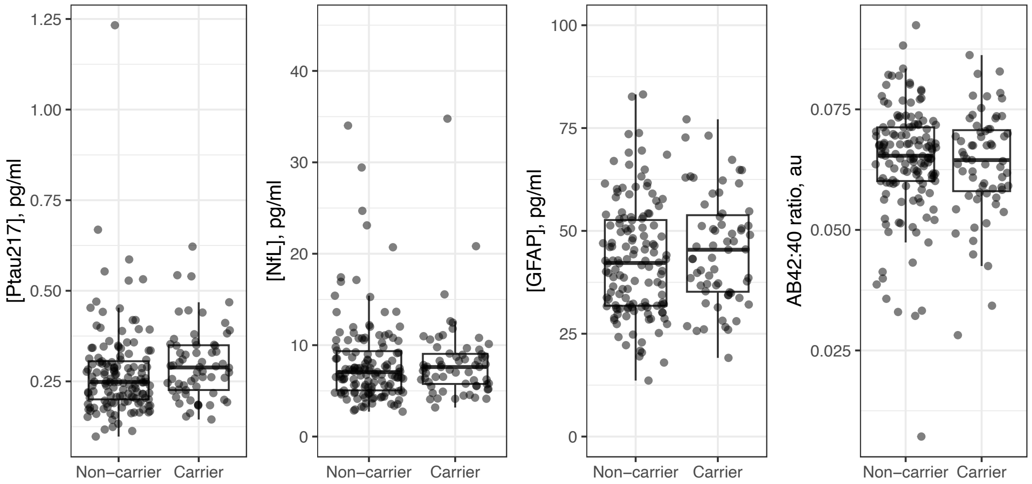

Supplementary Figure 2. APOE ε4 status and plasma biomarkers in ex-players *Boxplots show plasma biomarker concentrations, with median and interquartile ranges indicated, and individual data points plotted. Carriers show non-significant, numerically higher P-tau217, NfL, GFAP and lower AB42:40 ratios.*

Supplementary Tables

	**AD** (n=69)	**Older controls** (n=26)
Age at study visit (mean (SD))	75.45 (7.50)	76.58 (5.45)
Female (%)	36 (52.2)	13 (50.0)
MMSE (mean (SD))	20.83 (5.28)	29.00 (0.69)

Supplementary Table 1. Demographics of people with Alzheimer's disease and older healthy controls within the Minder / PCNoRAD Cohort
MMSE: mini mental status examination.

	**AD**	**Older Controls**
NFL, N	33	200
concentration, pg/ml, g.mean (g.sd)	30.1 (1.5)	18.9 (1.4)
GFAP, N	33	200
concentration, pg/ml, g.mean (g.sd)	213.7 (1.5)	127.2 (1.6)
P-tau217, N	33	199
concentration, pg/ml, g.mean (g.sd)	0.9 (1.9)	0.4 (1.5)
AB42:40, N	33	200
ratio, au, g.mean (g.sd)	0 (1.2)	0 (1.6)

Supplementary Table 2. Blood biomarker concentrations in people with Alzheimer's disease and older healthy controls within the Minder / PCNoRAD Cohort
NfL=neurofilament light; GFAP= glial fibrillary acidic protein; p-tau217= phospho-tau 217; AB42:40= amyloid beta 42:40 ratio; AD= Alzheimer's disease

	**NFL elevated**			**GFAP elevated**			**Ptau217 elevated**			**AB42:40 reduced**		
	Coef (* 95% CI)	***P***	***P.Corr***	***Coef (B,95% CI)***	***P***	***P.Corr***	***Coef (B,95% CI)***	***P***	***P.Corr***	***Coef (B,95% CI)***	***P***	***P.Corr***
RBANS												
Immediate memory	0.23 (-6.93-7.39)	0.949	1	-5.29 (-21.61-11.04)	0.524	1	-1.75 (-6.58-3.08)	0.475	1	0.38 (-7.15-7.91)	0.921	1
Visuo-construction	-4.98 (-10.04-0.09)	0.054	0.324	1.28 (-10.38-12.95)	0.829	1	-3.16 (-6.59-0.26)	0.07	0.42	0.59 (-4.79-5.96)	0.829	1
Language	0.33 (-5.03-5.7)	0.902	1	5.8 (-6.43-18.03)	0.351	1	-0.9 (-4.53-2.73)	0.626	1	-0.67 (-6.31-4.98)	0.816	1
Attention	-4.09 (-11.37-3.18)	0.269	1	9.58 (-7.02-26.18)	0.256	1	1.24 (-3.69-6.18)	0.62	1	7.35 (-0.25-14.95)	0.058	0.348
WAIS 4 Proc. Speed	-4.86 (-11.01-1.3)	0.121	0.726	3.88 (-10.23-18)	0.588	1	-3.38 (-7.54-0.79)	0.111	0.666	2.78 (-3.71-9.28)	0.399	1
WMS4 Auditory memory	-2.28 (-9.7-5.14)	0.545	1	-0.47 (-17.41-16.48)	0.957	1	-3.98 (-8.97-1.01)	0.117	0.702	0.45 (-7.36-8.25)	0.91	1
BDI	**4.04 (1.75-9.49)**	**0.001**	0.006	0.16 (0.03-0.95)	0.036	0.216	1.38 (0.75-2.54)	0.305	1	0.83 (0.34-2.03)	0.689	1
GAD7	**5.36 (2.24-13.01)**	**0**	0	0.14 (0.02-0.89)	0.031	0.186	0.87 (0.48-1.59)	0.654	1	0.89 (0.35-2.23)	0.807	1
Brief MI	1.56 (0.66-3.68)	0.304	1	0.45 (0.08-2.5)	0.335	1	1.73 (0.96-3.15)	0.07	0.28	0.84 (0.33-2.1)	0.706	1
Brief Bri	1.43 (0.64-3.21)	0.383	1	0.34 (0.05-2.09)	0.227	1	1.02 (0.57-1.84)	0.948	1	0.43 (0.15-1.2)	0.107	0.642
TES	1.55 (0.34-5.32)	0.519	0.519	2280277.81 (0-NA)	0.992	0.992	**2.81 (1.1-7.07)**	**0.027**	**0.027**	0.5 (0.03-2.67)	0.512	0.512

Supplementary Table 3. Blood biomarker changes and clinical phenotype in ex-players
*Coef.:coefficient, * Coefficient (ie. model beta) is shown for RBANS measures, WAIS4, WMS4 measures; but is shown exponentiated, as an odds ratio, for BDI, GAD7, Brief, and TES; CI:confidence interval; Bold typeface indicates significant P<0.05 after multiple comparisons correction. Model outcome measures are shown in the far left column, and predictors on the right.*

	Regression - years of play			Regression - concussion load (high vs low)			**Regression - years of play**, sensitivity analysis including 'washout' period in model		
	Coef (B,95% CI)	***P***	***P.Corr***	***Coef (B,95% CI)***	***P***	***P.Corr***	***Coef (B,95% CI)***	***P***	***P.Corr***
[NfL]	1 (0.98-1.02)	0.77	1.00	1.07 (0.91-1.26)	0.40	1.00	1.02 (0.99-1.05)	0.22	1.00
[GFAP]	1 (0.99-1.02)	0.75	1.00	1.15 (1-1.32)	0.05	0.20	1.01 (0.98-1.04)	0.37	1.00
[Ptau217]	1.01 (0.99-1.02)	0.40	1.00	0.98 (0.89-1.08)	0.71	1.00	1 (0.98-1.02)	0.96	1.00
[AB42:40]	1 (0.99-1.01)	0.50	1.00	0.98 (0.91-1.05)	0.58	1.00	1 (0.99-1.01)	0.84	1.00

Supplementary Table 4. Relationship of plasma biomarker concentrations with player career duration, and concussion load, in ex-players – sensitivity analysis including time since retirement *Coef.:coefficient; CI:confidence interval; the sensitivity analysis reflects a model in which a 'washout' period was included as a regressor, ie. the number of years between the ex-player's retirement and the study visit. Covariates included in the models included age and sex. Model outcome measures are shown in the far left column, and predictors on the right.*

	NFL elevated			GFAP elevated			Ptau217 elevated			AB42:40 reduced		
	Coef (95% CI)	*P*	*P.Corr*	*Coef (B,95% CI)*	*P*	*P.Corr*	*Coef (B,95% CI)*	*P*	*P.Corr*	*Coef (B,95% CI)*	*P*	*P.Corr*
RBANS												
Immediate memory	0.75 (-6.25-7.76)	0.833	1	-3.81 (-19.8-12.18)	0.639	1	-0.86 (-5.61-3.88)	0.72	1	-0.22 (-7.58-7.15)	0.954	1
Visuo-construction	-4.84 (-9.9-0.23)	0.061	0.366	1.72 (-9.95-13.38)	0.772	1	-2.96 (-6.41-0.49)	0.092	0.552	0.42 (-4.95-5.79)	0.878	1
Language	0.53 (-4.82-5.89)	0.845	1	6.39 (-5.81-18.58)	0.303	1	-0.57 (-4.21-3.07)	0.758	1	-0.9 (-6.52-4.73)	0.754	1
Attention	-3.81 (-11.05-3.44)	0.302	1	10.47 (-6.06-27)	0.213	1	1.77 (-3.16-6.71)	0.479	1	7.03 (-0.55-14.61)	0.069	0.414
WAIS 4 Proc. Speed	-4.4 (-10.41-1.61)	0.15	0.9	5.25 (-8.53-19.03)	0.454	1	-2.62 (-6.71-1.48)	0.209	1	2.25 (-4.09-8.6)	0.485	1
WMS4 Auditory memory	-1.53 (-8.59-5.53)	0.669	1	1.76 (-14.37-17.88)	0.83	1	-2.71 (-7.5-2.07)	0.264	1	-0.45 (-7.87-6.98)	0.906	1

Supplementary Table 5. Blood biomarker changes and clinical phenotype in ex-players (sensitivity analysis accounting for years of education) *Coef.:coefficient, * Coefficient (ie. model beta) is shown for RBANS measures, WAIS4, WMS4 measures; but is shown exponentiated, as an odds ratio, for BDI, GAD7, Brief, and TES; CI:confidence interval; Bold typeface indicates significant P<0.05 after multiple comparisons correction. Model outcome measures are shown in the far left column, and predictors on the right.*

	APOE E4 status		*Statistical test (regression)*	
	**Carriers**	**Non-Carriers**	*APOE term*	*Interaction - e4 carrier and ex-player*
**NFL concentration, pg/ml, g.mean (g.sd)**	7.54 (1.84)	7.71 (1.48)	B=1.17 (0.8-1.71), P=0.417 , Pcorr=1	B=0.85 (0.56-433), P=0.439 , Pcorr=1
**GFAP concentration, pg/ml, g.mean (g.sd)**	44.86 (1.76)	46.11 (1.4)	B=1.19 (0.84-1.68), P=0.336 , Pcorr=1	B=0.84 (0.57-1.22), P=0.35 , Pcorr=1
**P-tau217 concentration, pg/ml, g.mean (g.sd)**	0.25 (1.43)	0.27 (1.36)	B=1.09 (0.86-1.38), P=0.46 , Pcorr=1	B=1.01 (0.78-1.31), P=0.914 , Pcorr=1
**AB42:40 ratio, au, g.mean (g.sd)**	0.06 (1.29)	0.06 (1.22)	B=0.97 (0.82-1.15), P=0.752 , Pcorr=1	B=1.03 (0.86-1.23), P=0.785 , Pcorr=1

Supplementary Table 6. Fluid biomarkers and APOE ε4 status in all participants *NfL: neurofilament light; GFAP: glial fibrillary acidic protein. Covariates included in the models were age, and sex. Model outcome measures are shown in the far left column, and predictors on the right.*

	Group difference		Years of play		Concussion load (high vs low)		TES status (present vs absent)	
	Coef (B,95% CI)	***P***	*Coef (B,95% CI)*	***P***	*Coef (B,95% CI)*	***P***	*Coef (B,95% CI)*	***P***
**Region identified on group VBM (rugby vs control, in all participants)**								
Frontal/cingulate region	-108.7 (-207.3--10)	**0.031**	-8.2 (-16.5-0.2)	0.054	28.8 (-38.6-96.2)	0.4	-108.7 (-207.3--10)	**0.031**
**Regions related to years of play on VBM (rugby participants only)**								
Hippocampal GM region	9.5 (-37.9-56.9)	0.694	-7.7 (-11.6--3.9)	**<0.001**	6.3 (-25.8-38.4)	0.698	9.5 (-37.9-56.9)	0.694
White matter regions	-445.3 (-2824-1933.3)	0.712	-438.6 (-629--248.2)	**<0.001**	928.7 (-663.3-2520.7)	0.251	-445.3 (-2824-1933.3)	0.712
CSF regions	-767.9 (-3281.3-1745.4)	0.547	353.3 (147.4-559.2)	**0.001**	-250.7 (-1947.7-1446.4)	0.771	-767.9 (-3281.3-1745.4)	0.547

Supplementary Table 7. Regional brain volumes within VBM-identified regions – relationship to group, exposure, and clinical phenotype

VBM: voxel based morphometry; GM: grey matter; CSF: cerebrospinal fluid;; coef: coefficient; B:beta; CI: confidence interval. Covariates included in the models included age and sex. Model outcome measures are shown in the far left column, and predictors on the right.

	Years of play			Concussion load (high vs low)			TES status (present vs absent)		
	Coef (B,95% CI)	*P*	*P.Corr*	*Coef (B,95% CI)*	*P*	*P.Corr*	*Coef (B,95% CI)*	*P*	*P.Corr*
**Brain volume (continuous)**									
frontal lobe	-109.4 (-475.1-256.2)	0.56	1	267.3 (-2650.1-3184.7)	0.86	1	-1643.3 (-5994.4-2707.8)	0.457	1
temporal lobe	-51.7 (-302.4-199)	0.68	1	496.3 (-1471.9-2464.4)	0.62	1	1634.8 (-1342.3-4612)	0.28	1
hippocampal	-23.1 (-48.6-2.4)	0.08	0.48	-9.6 (-214.3-195.2)	0.93	1	-3.8 (-309.5-301.8)	0.98	1
parietal lobes	182.4 (-124.2-489)	0.24	1	549.5 (-1903.3-3002.2)	0.66	1	16.9 (-3646.6-3680.3)	0.993	1
occipital lobes	-195.1 (-379.1--11.1)	0.04	0.24	-815.6 (-2281.9-650.8)	0.27	1	-979.4 (-3190.1-1231.3)	0.383	1
ventricles	149.8 (-134.7-434.3)	0.3	1	-60.5 (-2333.7-2212.7)	0.96	1	1932.6 (-1453.1-5318.4)	0.262	1
**White matter FA (continuous)**									
corticospinal tract FA	0.0001 (-0.0005-0.0007)	0.72	1	0.0016 (-0.0029-0.0061)	0.49	1	0.002 (-0.004-0.009)	0.5	1
corpus callosum FA	0.0001 (-0.0007-0.0009)	0.79	1	-0.0034 (-0.0096-0.0029)	0.29	0.87	-0.005 (-0.014-0.005)	0.356	1
whole white matter FA	0.0001 (-0.0005-0.0006)	0.77	1	-0.0028 (-0.007-0.0015)	0.2	0.6	-0.003 (-0.01-0.003)	0.306	0.918

Supplementary Table 8. Imaging biomarkers and clinical phenotype in ex-players *Coef: coefficient; FA: diffusion tensor imaging fractional anisotropy; B: beta; CI: confidence interval. Covariates included in the models included age and sex in all cases, and total intracranial volume in the cases of the volumetric analyses. Model outcome measures are shown in the far left column, and predictors on the right.*

		Normalised brain volume						DTI FA		
		Frontal	Parietal	Hippocampal	Occipital	Temporal	Ventricular	Callosum	Corticospinal	Whole WM
**[NFL]**	r	**-0.21**	**-0.18**	-0.06	-0.16	-0.15	0.13	-0.06	-0.02	-0.06
	Pcorr	**0.010**	**0.044**	1.000	0.094	0.138	0.264	1.000	1.000	1.000
**[P-tau217]**	r	-0.09	0.04	**-0.19**	-0.03	0.04	0.12	-0.08	0.01	0.07
	Pcorr	0.927	1.000	**0.024**	1.000	1.000	0.332	1.000	1.000	1.000
**[GFAP]**	r	-0.15	**-0.19**	-0.08	-0.08	-0.04	0.15	-0.03	-0.07	-0.05
	Pcorr	0.119	**0.035**	0.985	1.000	1.000	0.146	1.000	1.000	1.000
**AB42:40 ratio**	r	0.09	-0.02	0.07	0.10	0.14	0.01	0.07	-0.07	0.03
	Pcorr	0.785	1.000	1.000	0.656	0.186	1.000	1.000	1.000	1.000

Supplementary Table 9. Spearman's correlations between plasma and imaging biomarkers in ex-players *DTI: diffusion tensor imaging; FA: fractional anisotropy; WM: white matter; NfL: neurofilament light; GFAP: glial fibrillary acidic protein;Pcorr: P value corrected for four fluid biomarkers.*

	OR (95% CI)	*P*	Pcorr
**Ptau217 elevation**	1.27 (0.58-2.66)	0.54	1
**NFL elevation**	0.82 (0.22-2.45)	0.738	1
**GFAP elevation**	0.15 (0.01-1.61)	0.126	0.504
**AB42:40 ratio reduction**	0.21 (0.01-1.07)	0.133	0.532

Supplementary Table 10. Relationship of cavum septum pellucidum to fluid biomarker abnormalities in ex-players *Logistic regression accounting for age and sex, within former rugby players. OR: odds ratio. NfL: neurofilament light; GFAP: glial fibrillary acidic protein. Model outcome measures are shown in the far left column, and predictors on the right.*
